# Stanford Type A Aortic Dissection During Adjuvant Capecitabine Chemotherapy for Colorectal Cancer

**DOI:** 10.1016/j.jaccas.2025.105362

**Published:** 2025-09-08

**Authors:** Yasuhiko Kawaguchi, Yuichiro Fukumoto, Chiaki Aichi, Kazuya Konakano, Mototsugu Tamaki, Hideki Kitamura, Yasuhide Okawa

**Affiliations:** Department of Cardiovascular Surgery, Nagoya Heart Center, Nagoya, Japan

**Keywords:** 5-FU, cancer, capecitabine, cardiovascular toxicity, chest pain, dissection

## Abstract

**Background:**

Capecitabine, an oral prodrug of 5-fluorouracil, is widely used for gastrointestinal malignancies. While its coronary toxicity is well documented, large-vessel complications such as aortic dissection are rarely reported.

**Case Summary:**

We present a 65-year-old man with colorectal cancer who developed Stanford type A aortic dissection 3 days after initiating adjuvant capecitabine therapy. Imaging revealed extensive dissection from the ascending to the abdominal aorta. Emergency hemiarch replacement was successfully performed.

**Discussion:**

The absence of prior cardiovascular disease, close temporal relationship, and known vasotoxicity of fluoropyrimidines suggest a likely association. Fluoropyrimidines may induce endothelial injury, vasospasm, and hypertension. A history of smoking in our patient may have contributed to vascular susceptibility.

**Take-Home Messages:**

Capecitabine, though well tolerated, may rarely cause life-threatening vascular complications such as aortic dissection. Clinicians should maintain a high index of suspicion for atypical vascular symptoms during therapy.

## Graphical Abstract

**Figure:**
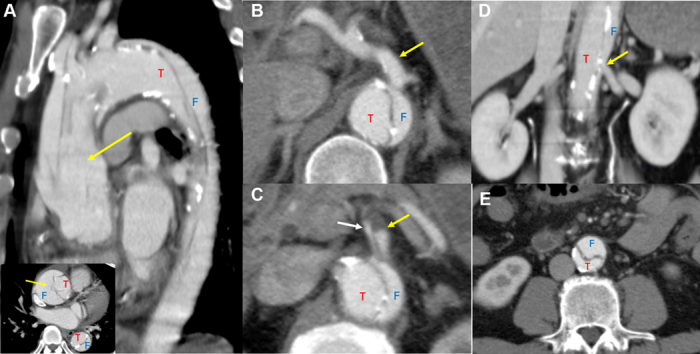

## History of Presentation

A 65-year-old man presented with sudden-onset abdominal and back pain. On arrival, his blood pressure was 170/89 mm Hg, with a notable difference between upper extremities. His heart rate was 82 beats/min, respiratory rate was 16 breaths/min, and oxygen saturation level was 98% on room air.

## Past Medical History

The patient had no known cardiovascular risk factors aside from a history of smoking. Two months prior, he had undergone laparoscopic high anterior resection for synchronous colorectal cancers. Histology revealed moderately differentiated adenocarcinomas of the sigmoid colon and rectum, classified as stage IIa (pT3N0M0, Union for International Cancer Control 8th edition), with lymphovascular invasion and sampling of <12 lymph nodes. Adjuvant chemotherapy with oral capecitabine (1,250 mg/m^2^ twice daily) had been initiated 3 days before presentation.^1^

## Differential Diagnosis

Initial considerations included myocardial infarction, acute pancreatitis, nephrolithiasis, gallstone disease, and capecitabine-induced abdominal side effects.

## Investigations

**Figure 1:**
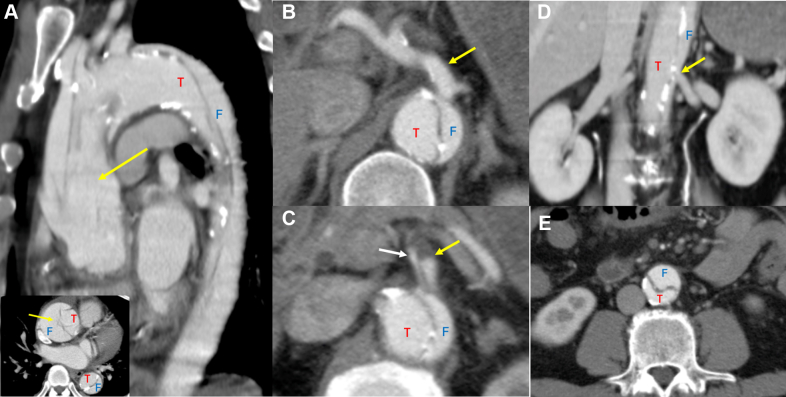
Computed Tomography Angiography at Presentation (A) Intimal tear (arrow) identified in the dissected ascending aorta with extension into the innominate artery. (B) Celiac artery perfused via the false lumen (arrow). (C) Superior mesenteric artery shows compression of the true lumen (white arrow) by a thrombosed false lumen (yellow arrow). (D) Left renal artery perfused by the true lumen (arrow). (E) True lumen compressed by the false lumen at the level of the distal abdominal aorta. F = false lumen; T = true lumen.

Electrocardiogram showed normal sinus rhythm without ST-segment changes. Contrast-enhanced computed tomography angiography (CTA) revealed a Stanford type A acute aortic dissection (AAD) extending from the ascending aorta through the thoracic and abdominal aorta. A patent false lumen was visualized, with the primary intimal tear located in the ascending aorta. Dissection involvement included the innominate artery, celiac trunk, superior mesenteric artery, and left renal artery (Figure 1). Sporadic intimal calcification was seen throughout the aorta (Video 1).

## Management

**Figure 2:**
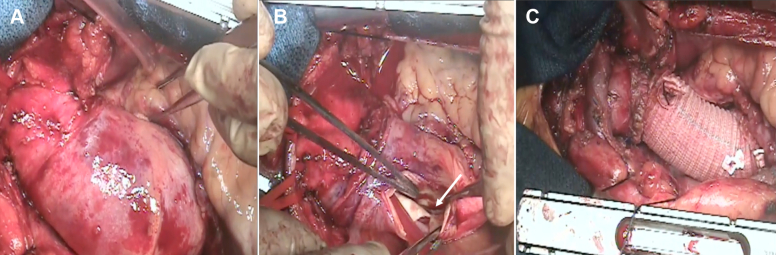
Intraoperative Findings (A) Dissected ascending aorta visualized after pericardiotomy. (B) Intimal tear (arrow) identified in the mid ascending aorta under circulatory arrest. (C) Ascending aorta and proximal arch replaced with a 28-mm Dacron graft.

The patient underwent emergent surgical repair via median sternotomy under general anesthesia. Cardiopulmonary bypass was established through the femoral artery and right atrium. Deep hypothermic circulatory arrest and selective antegrade cerebral perfusion were initiated. The entry tear was identified in the mid ascending aorta. Hemiarch replacement was performed using a 28-mm Dacron graft (Figure 2, Video 2). The surgery was completed without intraoperative complications.

## Outcome and Follow-Up

**Figure 3:**
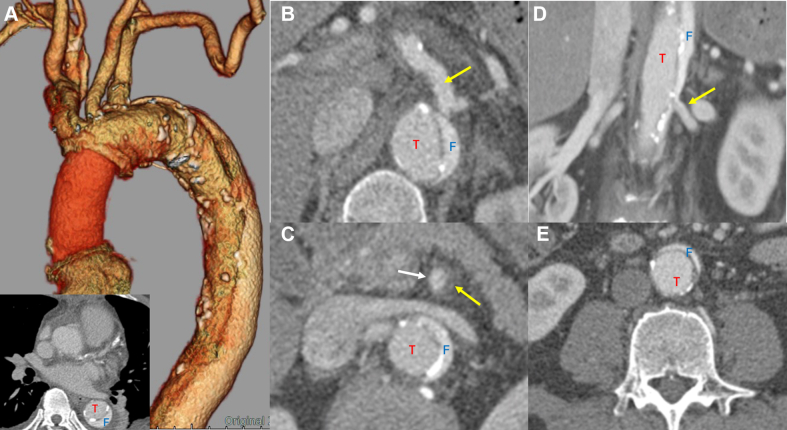
Postoperative Computed Tomography Angiography (A) Three-dimensional reconstruction demonstrating graft replacement of the ascending aorta and axial image at the level of the mid ascending aorta. (B) Stable anatomy of the celiac artery (arrow). (C) Re-expansion of the superior mesenteric artery true lumen (white arrow) with regression of the false lumen (yellow arrow). (D) Persistent perfusion of the left renal artery without new complications (arrow). (E) Dilated true lumen and regressed false lumen at the level of the distal abdominal aorta. F = false lumen; T = true lumen.

The patient was extubated on the same day and was transferred out of the intensive care unit on postoperative day 1. He was discharged home on postoperative day 9 with optimized antihypertensive therapy. Follow-up CTA confirmed a residual dissection from the aortic arch to the abdominal aorta, without signs of malperfusion or graft-related complications (Figure 3, Video 3). Annual surveillance CTA has shown a stable aortic diameter (maximum diameter: 46 mm), with the true and false lumen measuring 30 and 16 mm, respectively. At the 5-year follow-up, he remained asymptomatic, without need for aortic reintervention or colorectal cancer recurrence.

## Discussion

Fluoropyrimidines such as 5-fluorouracil (5-FU) and its oral prodrug capecitabine are widely used in treating gastrointestinal and breast cancers. These antimetabolites exert their anticancer effects by disrupting DNA and RNA synthesis. Though generally well tolerated, these agents are associated with cardiovascular toxicity, including angina, arrhythmias, heart failure, and, rarely, sudden cardiac death. The proposed mechanisms include coronary vasospasm, oxidative stress, and direct endothelial injury.^2^ Capecitabine undergoes enzymatic conversion to 5-FU, with the final activation step occurring predominantly in tumor tissues owing to high thymidine phosphorylase expression. This improves tumor selectivity and tolerability. However, at the cellular level, capecitabine's active metabolite is functionally identical to 5-FU, and it retains the same potential for vascular toxicity.^2^

Although the angiogenesis inhibitors targeting the vascular endothelial growth factor (VEGF) pathway, VEGF inhibitors such as bevacizumab, sunitinib, and sorafenib, are more commonly associated with AAD owing to vasoconstriction and endothelial dysfunction,^3^ fluoropyrimidines have also been implicated in noncoronary vascular events, including AAD, in addition to their known cardiotoxic effects. AAD linked to capecitabine monotherapy is exceedingly rare; in one report, Sclafani et al^4^ described a patient who developed abdominal aortic dissection shortly after capecitabine administration, with histologic evidence of vascular damage. Of note, combined regimens of VEGF inhibitors and 5-FU are commonly used for synergistic antitumor efficacy, but this may also enhance vascular toxicity.^5^ A case of AAD during combination therapy of sorafenib, gemcitabine, and capecitabine has been reported.^6^

Capecitabine-based regimens have been associated with acute aortic thrombosis,^7^ and spontaneous coronary artery dissection has also been reported during 5-FU therapy. The combination of hemodynamic change due to vomiting, chemotherapy-induced vasospasm, and endothelial injury has been considered as contributing factor for AAD.^8^ These cases suggest a broader spectrum of fluoropyrimidine-induced vascular injury beyond known cardiotoxicity.

In our patient, no major cardiovascular risk factors were present aside from smoking. A recent meta-analysis indicates that smokers receiving fluoropyrimidines may have increased cardiovascular risk, even in the absence of overt atherosclerosis; this is likely owing to endothelial dysfunction, vasospasm, and inflammation.^9^

Interestingly, our patient exhibited sporadic intimal calcification throughout the aorta on CTA. Prior research has shown that patients with AAD have significantly greater calcification in the ascending aorta and arch compared with controls, suggesting a possible role for increased aortic stiffness in dissection risk.^10^ Moreover, although our patient did not receive radiotherapy, it is worth noting that fluoropyrimidines are often used concurrently with radiation given their radiosensitizing effects. Although not clearly established, radiation-induced vascular calcification may further contribute to long-term aortic vulnerability.

We hypothesize that in this case, capecitabine-induced vascular toxicity—possibly potentiated by smoking history and underlying aortic calcification—led to endothelial damage and acute hypertension, precipitating Stanford type A AAD. While a direct causal link cannot be confirmed, the close temporal relationship and the absence of other strong risk factors support a likely association.

After hemiarch repair, the patient was managed conservatively for residual distal dissection. Annual surveillance with CTA demonstrated stability of the dissected aorta, with no evidence of aneurysmal progression or branch vessel compromise. Given the stable anatomy and absence of symptoms, continued annual imaging follow-up remains the standard of care.

## Conclusions

Capecitabine monotherapy, though widely used and well tolerated in colorectal cancer, may cause rare but severe vascular complications such as aortic dissection. This case highlights a rare but life-threatening vascular complication of capecitabine therapy. Clinicians should maintain a high index of suspicion for acute aortic events in patients on fluoropyrimidines presenting with chest, back, or abdominal pain, even in the absence of known cardiovascular disease.

## Funding Support and Author Disclosures

The authors have reported that they have no relationships relevant to the contents of this paper to disclose.

**Take-Home Messages**

• Fluoropyrimidines, including capecitabine, are associated with rare but serious vascular toxicity beyond well-known coronary events.
• Aortic dissection can present early during treatment and may mimic other abdominal or cardiac emergencies. Prompt diagnosis with CTA and multidisciplinary management are essential for survival.

## Appendix

Video 1 Preoperative Computed Tomography Angiography (Cine Loop) Aortic dissection extending from the ascending aorta to the terminal aorta, involving the innominate artery, celiac artery, superior mesenteric artery (SMA), and left renal artery. SMA shows compression of the true lumen by a thrombosed false lumen, with maintained distal perfusion. Sporadic calcification was seen throughout the aorta.

Video 2 Operative Procedure Surgical repair via median sternotomy. The entry tear in the mid ascending aorta was resected, and hemiarch replacement was performed using a 28-mm Dacron graft under deep hypothermic circulatory arrest with selective antegrade cerebral perfusion.

Video 3 Postoperative Computed Tomography Angiography (Cine Loop) The ascending aorta to proximal arch was replaced with a Dacron graft. Residual distal dissection is seen beyond the anastomosis. The descending aorta demonstrates expansion of the true lumen, with regression of the false lumen in the superior mesenteric artery.

## References

[1] Twelves C.J. (2006). Xeloda® in Adjuvant Colon Cancer Therapy (X-ACT) trial: overview of efficacy, safety, and cost-effectiveness. Clin Colorectal Cancer.

[2] Sara J.D., Kaur J., Khodadadi R. (2018). 5-fluorouracil and cardiotoxicity: a review. Ther Adv Med Oncol.

[3] Oshima Y., Tanimoto T., Yuji K., Tojo A. (2017). Association between aortic dissection and systemic exposure of vascular endothelial growth factor pathway inhibitors in the Japanese adverse drug event report database. Circulation.

[4] Sclafani F., Carnaghi C., Colombo P. (2010). Case report of acute aortic dissection during treatment with capecitabine for a late recurrence of breast cancer. Chemotherapy.

[5] Cameron A.C., Touyz R.M., Lang N.N. (2016). Vascular complications of cancer chemotherapy. Can J Cardiol.

[6] Serrano C., Suárez C., Andreu J., Carles J. (2010). Acute aortic dissection during sorafenib-containing therapy. Ann Oncol.

[7] Wong H.C.Y., Lim F.M.Y., Cheng A.C.K. (2019). Acute aortic thrombosis related to adjuvant capecitabine-oxaliplatin in a Chinese patient with resected adenocarcinoma of sigmoid colon. Arch Clin Med Case Rep.

[8] Abbott J.D., Curtis J.P., Murad K. (2003). Spontaneous coronary artery dissection in a woman receiving 5-fluorouracil: a case report. Angiology.

[9] Li C., Ngorsuraches S., Chou C., Chen L., Qian J. (2021). Risk factors of fluoropyrimidine induced cardiotoxicity among cancer patients: a systematic review and meta-analysis. Crit Rev Oncol Hematol.

[10] Yang C.J., Tsai S.H., Wang J.C. (2019). Association between acute aortic dissection and the distribution of aortic calcification. PLoS One.

